# Anti-Inflammatory Activity of the Constituents from the Leaves of *Perilla frutescens* var. *acuta*

**DOI:** 10.3390/ph16121655

**Published:** 2023-11-28

**Authors:** Isoo Youn, Sujin Han, Hee Jin Jung, Sang Gyun Noh, Hae Young Chung, Yean Kyoung Koo, Sunhye Shin, Eun Kyoung Seo

**Affiliations:** 1 Graduate School of Pharmaceutical Sciences, College of Pharmacy, Ewha Womans University, Seoul 03760, Republic of Korea; isooyoun87@gmail.com (I.Y.); sujinh94@gmail.com (S.H.); 2 Department of Pharmacy, College of Pharmacy, Pusan National University, Busan 46241, Republic of Korea; hjjung2046@pusan.ac.kr (H.J.J.); rskrsk92@pusan.ac.kr (S.G.N.); hyjung@pusan.ac.kr (H.Y.C.); 3 Department of R&I Center, COSMAXBIO, Seongnam 13487, Republic of Korea; ygkoo@cosmax.com; 4 Major of Food and Nutrition, Division of Applied Food System, Seoul Women’s University, Seoul 01797, Republic of Korea

**Keywords:** *Perilla frutescens* var. *acuta*, anti-inflammation, peroxisome proliferator-activated receptor, nuclear factor kappa B, flavonoid diglucuronide

## Abstract

*Perilla frutense* var. *acuta* (Lamiaceae) has been used to treat indigestion, asthma, and allergies in traditional medicine. In this study, luteolin 7-*O*-diglucuronide (**1**), apigenin 7-*O*-diglucuronide (**2**), and rosmarinic acid (**3**) were isolated from the leaves of *P. frutescens* var. *acuta* through various chromatographic purification techniques. Several approaches were used to investigate the anti-inflammatory activity of the constituents (**1**–**3**) and their working mechanisms. In silico docking simulation demonstrated that **1**–**3** would work as a PPAR-α/δ/γ agonist, and in vitro PPAR-α/δ/γ transcriptional assay showed that the *Perilla* water extract (PWE) and **3** increased PPAR-α luciferase activity (1.71 and 1.61 times of the control (PPAR-α + PPRE, *p* < 0.001)). In the NF-κB luciferase assay, **1** suppressed NF-κB activity the most (56.83% at 5 µM; 74.96% at 10 µM; 79.86% at 50 µM). In addition, **1** and **2** inhibited the mRNA expression of NF-κB target genes, including *Il6*, *Mcp1*, and *Tnfa*, at 50 µM, and **3** suppressed the genes at the mRNA level in a dose-dependent manner. We report that **1** and **2** exert anti-inflammatory effects through NF-κB inhibition, and the PPAR-α/NF-κB signaling pathway is related to the anti-inflammatory activity of **3**.

## 1. Introduction

*Perilla frutescens* (L.) Britton var. *acuta* Kudo, which belongs to the Lamiaceae family, is native to south and east Asia and is widely cultivated in Korea, China, India, Japan, and Vietnam as a functional food, a spice, and the primary source of perilla oil [1,2]. While *P. frutescens* var. *acuta* has been known to treat indigestion, fever, asthma, and anxiety in traditional medicine [2], modern scientists have also reported anti-allergic, antimicrobial, antioxidant, and cytotoxic activities of *P. frutescens* var. *acuta* [3]. This plant contains various compounds, including fatty acids, flavonoids, phenolic acids, triterpenoids, and volatile oils [3]. Although previous studies have reported that *P. frutescens* shows anti-inflammatory effects through the inhibition of tumor necrosis factor-α (TNF-α), cyclooxygenase-2 (COX-2), interleukin-6 (IL-6), IL-8, and inducible nitric oxide synthase (iNOS), the active ingredients and their mechanisms of action are not well understood yet [4,5].

Inflammation is a coordination of multiple signaling pathways to regulate the inflammatory mediators from the blood when harmful stimuli, including pathogens, damaged cells, or irritants, invade the body [6]. Although the inflammatory response is essential to protect the body, excessive or chronic inflammation can be related to non-communicable diseases (NCD), including cardiovascular diseases, cancer, and diabetes. A Global Burden of Disease study has reported that NCD contributed to 50.7% of total deaths worldwide in 2007, and the number increased to 73.4% in 2017 [7]. As ongoing studies have been conducted to elucidate the pathology of irregular inflammation, peroxisome proliferator-activated receptor (PPAR) and nuclear factor kappa B (NF-κB) have been found to be the transcription factors related to the inflammatory responses [8]. PPARs inhibit NF-κB binding to DNA promoter regions and its target gene expression [9]. Thus, PPAR agonists can suppress the release of pro-inflammatory cytokines/chemokines, such as IL-6, monocyte chemoattractant protein-1 (MCP-1), and TNF-α [10].

Natural products (NP) can be a valuable source to treat inflammatory diseases, owing to NP’s various pharmacological activities and low toxicity [11]. For example, *Lavandula viridis* L’Hér. essential oil has been investigated for its anti-inflammatory activity and was found to inhibit the release of nitric oxide (NO), iNOS, and COX-2 by suppressing the NF-κB pathway [12]. The flavonol-enriched *Uvaria alba* extract has also been shown to down-regulate proteins and the mRNA expression of iNOS, COX-2, TNF-α, IL-1β, and IL-6 by blocking the NF-κB p65 subunit and, thus, inhibiting NF-κB activation in RAW 264.7 cells [13].

In this study, luteolin 7-*O*-diglucuronide (**1**), apigenin 7-*O*-diglucuronide (**2**), and rosmarinic acid (**3**) were isolated from the leaves of *P. frutescens* var. *acuta*. The anti-inflammatory activity of **1**–**3** was investigated through in silico docking simulation against PPAR-α/δ/γ, in vitro PPAR-α/δ/γ transcriptional luciferase assay, NF-κB luciferase assay, and measurement of NF-κB target gene expression.

## 2. Results

### 2.1. Isolation of the Compounds

**Figure 1:**
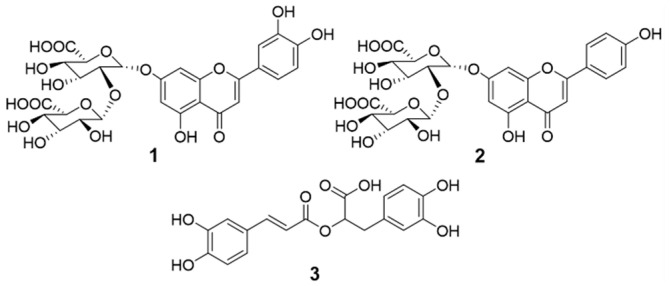
Structures of the isolated compounds (**1**–**3**) in this study.

Compounds **1**–**3** were isolated from the *Perilla* water extract (PWE) using various column chromatography techniques. Identification of structures was conducted based on 1D/2D NMR data (^1^H, ^13^C, DEPT 135, HSQC, COSY, HMBC, and NOESY spectra), optical rotation, UV, and HR-MS analyses. The isolated compounds were, thus, confirmed as luteolin 7-*O*-diglucuronide (**1**), apigenin 7-*O*-diglucuronide (**2**), and rosmarinic acid (**3**) (Figure 1). **1** and **2** were first isolated from the *Elodea* Species [14], and later they were also found in *P. frutescens* var. *acuta* [15,16]. **1** and **2** belong to the flavonoids, one of the abundant secondary metabolites in *P. frutescens* [17]. Apigenin and its derivatives have been known as one of the main flavonoids in this species [17]. Compound **3** has been isolated from *Rosmarinus officinalis* L. for the first time and was also found in the leaves of *P. frutescens* var. *acuta* in 1982 [18]. **3** is generally found in the Boraginaceae and Lamiaceae families [19].

### 2.2. In Silico Docking Simulation

In silico molecular docking simulation shows the interaction of the receptor and ligand in a preferred position with the minimum free binding energy for a stable complex and helps to predict the action of the ligand as an agonist or antagonist [20]. In this docking study, the non-covalent interactions were shown between the isolated compounds and PPAR-α/δ/γ. Complexes with lower energies are more stable in simulation work [21].

**Figure 2:**
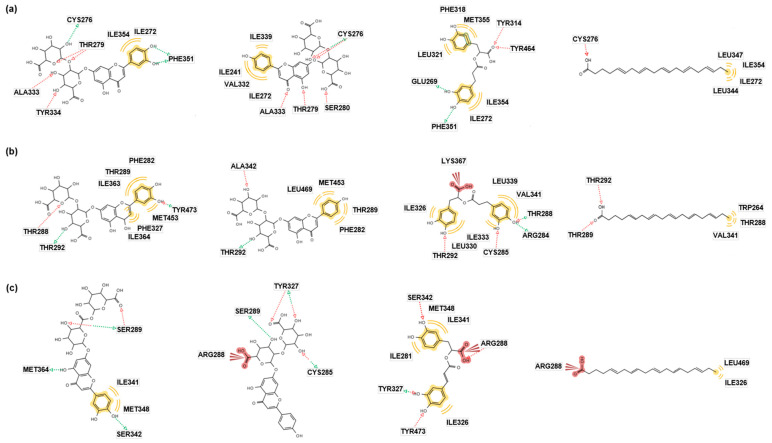
Docking simulations of **1**, **2**, **3**, and EPA against (**a**) PPAR-α, (**b**) PPAR-δ, and (**c**) PPAR-γ in AutoDock 4.2. The green arrow indicates the hydrogen bond (H-bond) donor, the red arrow indicates the H-bond acceptor, and the yellow color indicates the hydrophobic interaction or van der Waals force. (ALA, Alanine; ARG, Arginine; CYS, Cysteine; GLU, Glutamic acid; ILE, Isoleucine; LEU, Leucine; LYS, Lysine; MET, Methionine; PHE, Phenylalanine; SER, Serine; THR, Threonine; TRP, Tryptophan; TYR, Tyrosine; VAL, Valine).

The docking energies of **1**–**3** with PPAR-α were lower than that of the control (eicosapentaenoic acid, EPA) or equal to those in Autodock Vina, Autodock 4, and Dock 6 (Table 1). In particular, **1** (−13.2 Kcal/mol) showed the highest affinity for the binding site in Autodock 4, and **3** consistently showed modest binding affinity in the three systems. We also investigated the pharmacophores contributing to the interactions of the ligands and PPAR-α. Figure 2a demonstrates molecular docking models of **1**–**3** and EPA (a pan-activator of PPARs) [22]. The green arrow indicates the hydrogen bond (H-bond) donor, the red color indicates the H-bond acceptor, and the yellow color indicates the hydrophobic interaction or van der Waals force. In PPAR-α, **1** and **2** showed H-bonds with the residue CYS^276^, which commonly interacts with EPA. Although **3** did not have the same H-bond with EPA, two common residues, ILE^272^ and ILE^354^, produced hydrophobic interactions with both **3** and EPA. These results indicated that **1**–**3** may have pharmacological actions similar to the control, EPA.

The interactions between **1**–**3** and PPAR-δ were more stabilized than that of EPA in Autodock Vina, Autodock 4, and Dock 6 (Table 1). In addition, the best docking poses of the ligands with PPAR-δ were investigated (Figure 2b). In PPAR-δ, **1**–**3** formed a H-bond with the residue THR^292^, which also interacted with EPA. Because an interacting residue affects the binding mode of protein and ligand, **1**–**3** may show similar binding modes with EPA, suggesting that **1**–**3** may present PPAR-δ agonistic activity.

**Figure 3:**
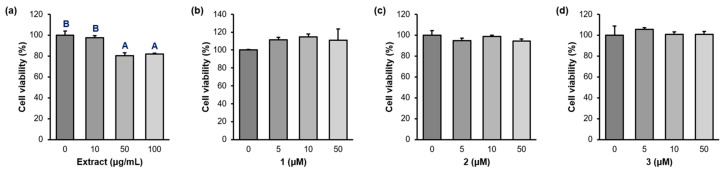
Cell viability of the *P. frutense* var. *acuta* extract (PWE) and isolated compounds **1**–**3**. (**a**) PWE (0, 10, 50, 100 µg/mL). (**b**–**d**) **1**–**3** (0, 5, 10, 50 µM). Raw 264.7 cell line was treated with the indicated doses of the drugs for 24 h, and cell viability was measured using MTT assay. ^A,B^ Different superscripts show critical differences at *p* < 0.05 using Duncan’s multiple comparison test. If a group has the same superscript as another group, it indicates that they are not statistically different.

Compounds **1** and **2** showed more stable binding affinities with PPAR-γ than the control (EPA) in Autodock 4 and Dock6 (Table 1). Moreover, **3** demonstrated higher docking energies to the PPAR-γ receptor than the control in all three systems. In pharmacophore analysis (Figure 2c), **2** and **3** generated an ionic bond with the residue ARG^288^, which also formed an interaction with EPA. Because an ionic bond is the strongest interaction between a ligand and molecule, it significantly influences the binding mode between the protein and ligand. Therefore, **2** and **3** may also show PPAR-γ agonistic activity. Although **1** did not show any common interaction with EPA, it formed additional H-bonds with the receptor compared to the control. Therefore, **1** may be a promising agonist candidate for PPAR-γ.

These results demonstrate that compounds **1**–**3** have several interactions with the residues in PPAR-α, PPAR-δ, and PPAR-γ. These residues also interact with the control (EPA), and, thus, **1**–**3** would show pan-agonistic actions for PPAR.

In a further study, we performed an in silico pharmacokinetic study through the ADMET prediction of **1**–**3** to evaluate their bioavailability and toxicity (Table 2) [23]. Compounds **1** and **2** showed high molecular weights and a large number of H-bond acceptors and donors, which caused the rejection of the Lipinski rule. However, MCE-18 and the Pfizer rule indicated that **1** and **2** possess high drug-likeness. Moreover, these compounds showed low F_20%_, which means optimal bioavailability and distribution compared to the control. Toxicity profiling showed a low possibility of hepatotoxicity, carcinogenicity, and respiratory toxicity. Compound **3** displays several physicochemical properties which support the suitability of **3** as a drug. In addition, it was accepted according to the Lipinski, Pfizer, and GSK rules. Although **3** showed risk of hepatotoxicity and carcinogenicity, it was predicted to be non-toxic in the other toxicity sections. Taken together, **1**–**3** can be effective PPAR agonists despite some drawbacks.

### 2.3. Cell Viability

As cytotoxicity is a critical obstacle for activity study, the cytotoxicity of the compounds was examined before performing the in vitro assays. Raw 264.7 macrophages were treated with PWE (0, 10, 50, 100 µg/mL) and the compounds (0, 10, 50, 100 µM) for 24 h, and cell toxicity was determined using an MTT assay (Figure 3 and Table S2). Although PWE showed 80.3 and 81.8% cell viability at 50 and 100 µg/mL concentrations, the cell viability of **1**–**3** exceeded 94% in all concentrations against Raw 264.7 cells.

### 2.4. PPAR-α/δ/γ Transcriptional Activity

**Figure 4:**
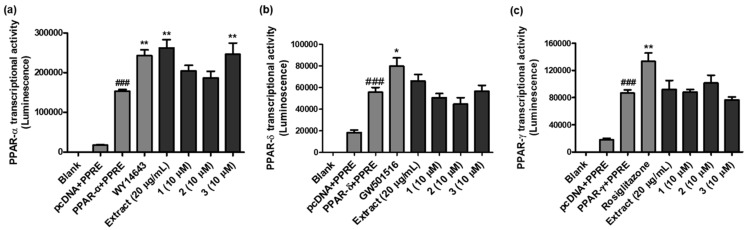
PPAR-α/δ/γ transcriptional activities of PWE and **1**–**3** from *P. frutescens* var. *acuta*. (**a**) The effect of PWE and **1**–**3** on PPAR-α transcriptional activity was measured with the PPRE luciferase system. ^###^ *p* < 0.001 vs. pcDNA + PPRE group, ** *p* < 0.001 vs. PPAR-α + PPRE group. (**b**) The effect of PWE and **1**–**3** on PPAR-δ transcriptional activity was evaluated through the PPRE luciferase system. ^###^ *p* < 0.001 vs. pcDNA + PPRE group. * *p* < 0.05 vs. PPAR-δ + PPRE group. (**c**) The effect of PWE and **1**–**3** on PPAR-γ transcriptional activity was measured using the PPRE luciferase system. ^###^ *p* < 0.001 vs. pcDNA + PPRE group. ** *p* < 0.005 vs. PPAR-γ + PPRE group.

Compounds **1**–**3** (10 µM each) were submitted to in vitro PPAR-α/δ/γ transactivation potency testing in Ac2F cells to verify the validity of the results from the docking simulation. As shown in Figure 4a and Table S3, WY14643 (a well-known PPAR-α agonist), PWE and **3** increased PPAR-α promoter luciferase reporter activity (1.58, 1.71, and 1.61 times, respectively; *p* < 0.001) compared to the control (PPAR-α + PPRE group); PWE and **3** showed higher PPAR-α agonistic activity than WY14643. The in silico docking analysis also suggested that **3** had the strongest binding affinity as a PPAR-α agonist in Autodock Vina and Dock6.

PPAR-α regulates fatty acid catabolism and ketogenesis and has been known to be significantly involved in inflammation. One of the underlying mechanisms of PPAR-α for anti-inflammation is the involvement of NF-κB. The binding of PPAR-α with the NF-κB p65 and JNK-responsive part of c-JUN inhibits IL-6 production [24], and the formation of the complex comprised of PPAR-α, sirtuin 1 (SIRT1), and NF-κB p65 deacetylates the p65 subunit, suppressing pro-inflammatory chemokines like MCP-1 in cardiomyocytes [25]. The catabolism of leukotriene B_4_ (LTB_4_) by PPAR-α is another critical mechanism of anti-inflammation [26]. LTB_4_ is a potent chemotactic agent that can induce inflammation, and PPAR-α can activate the enzyme (acyl-CoA oxidase) for LTB_4_ catabolism to inhibit the inflammatory response.

As shown in Figure 4b and Table S4, the PPAR-δ transcriptional activities of PWE and the compounds were compared with that of GW501516 (a widely used PPAR-δ agonist). However, none of them showed higher potency than GW501516 (1.43 times more potent than the control (PPAR-δ + PPRE), *p* < 0.05). The order of the in silico binding energies between the ligands and PPAR-δ did not precisely match the in vitro PPAR-δ transcriptional potency.

The PPAR-γ transactivation activity was also measured with PWE and the compounds using Ac2F cells transiently transfected with pcDNA/PPAR-γ and PPRE (Figure 4c and Table S5). Although the PPAR-γ agonistic potency of **2** was 1.17 times stronger than that of the control (PPAR-γ + PPRE), it did not exceed the activity of rosiglitazone (a well-known PPAR-γ agonist; 1.54 times stronger than the control).

### 2.5. NF-κB Transcriptional Activity

**Figure 5:**
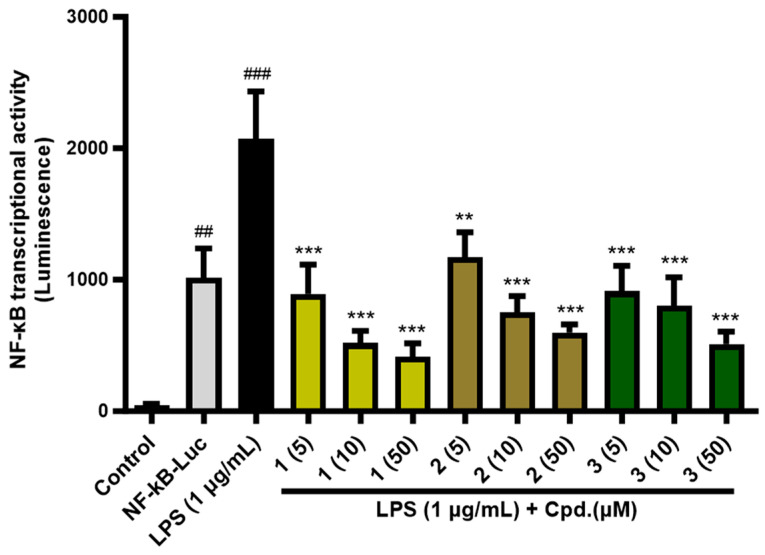
NF-κB transcriptional activity of **1**–**3** (5, 10, 50 µM) from *P. frutense* var. *acuta*. NF-κB transcriptional activity was evaluated with LPS-treated HEK293T cells with or without **1**–**3** treatment. ^###^ *p* < 0.001 and ^##^ *p* < 0.01 vs. control, *** *p* < 0.001 and ** *p* < 0.01 vs. LPS-treated group.

Previous studies have reported that PPAR-α/δ/γ inhibit NF-κB activation involved in inflammatory responses [27,28,29]. Therefore, we investigated whether the compounds could inhibit inflammation via NF-κB signal transduction. As demonstrated in Figure 5 and Table S6, NF-κB-driven luciferase assay was performed using HEK293T cells, and the NF-κB transcriptional activity remarkably increased after 1 µg/mL of lipopolysaccharide (LPS) induction for 6 h. On the other hand, compound treatment (5, 10, 50 µM) suppressed the NF-κB transcriptional activity in a dose-dependent manner and **1** (% inhibition: 56.8% at 5 µM; 75.0% at 10 µM; 79.9% at 50 µM) showed the strongest activity, followed by **3** (% inhibition: 43.3% at 5 µM; 63.6% at 10 µM; 71.1% at 50 µM) and **2** (% inhibition: 55.9% at 5 µM; 61.2% at 10 µM; 75.3% at 50 µM). Compound **3** has previously been shown to down-regulate the PPAR-γ/NF-κB-mediated signaling pathway in rat myocardial tissue [30].

### 2.6. NF-κB Target Gene Expression

**Figure 6:**
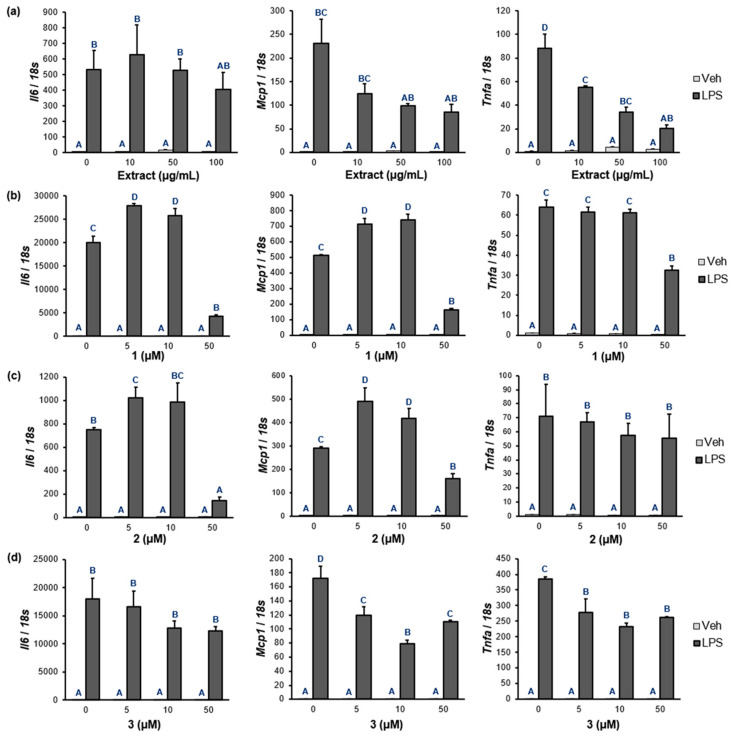
The effects of PWE and **1**–**3** on the mRNA levels of pro-inflammatory cytokines in Raw 264.7 cells pretreated with LPS. (**a**) PWE (0, 10, 50, 100 µg/mL). (**b**–**d**) **1**–**3** (0, 5, 10, 50 µM). Raw 264.7 cells were pretreated with each component dissolved in DMSO for 24 h, and then 100 ng/mL of LPS for 6 h. The mRNA levels were quantified using quantitative real-time polymerase chain reaction with normalization relative to 18s rRNA. Data are presented as fold changes compared to Veh-treated control. ^A–D^ Different superscripts mean remarkable differences at *p* < 0.05 by Duncan’s multiple comparison test. If a group has an identical superscript with another group, it indicates that they are statistically the same. (Veh-treated control = 1; means ± standard error of the mean; *n* = 3). (Veh, vehicle; *Il6*, interleukin 6; Mcp1, monocyte chemoattractant protein 1; *Tnfa*, tumor necrosis factor α).

Based on the PPAR-α/δ/γ and NF-κB luciferase assays of **1**–**3**, the expression levels of the NF-κB target genes were measured for *Il6*, *Mcp1*, and *Tnfa* in Raw 264.7 macrophages. As shown in Figure 6a and Table S7, PWE significantly inhibited the mRNA expression of *Il6*, *Mcp1*, and *Tnfa* in a dose-dependent manner. In the case of **1** and **2**, they notably inhibited the mRNA levels of *Il6* (% inhibition: **1**, 79.0%; **2**, 81.0%) and *Mcp1* (% inhibition: **1**, 67.9%; **2**, 44.7%) at 50 µM (Figure 6b,c and Table S7). The mRNA levels of *Mcp1* and *Tnfa* treated with **3** were strongly inhibited at a concentration of 10 µM (% inhibition: *Mcp1*, 53.9%; *Tnfa*, 39.6%).

The anti-inflammatory potency and efficacy of the tested compounds can be evaluated based on the IC_50_ value of positive controls. For example, a well-known anti-inflammatory natural compound, quercetin, has shown an IC_50_ value of 10 μM for the inhibition of TNF-α production [31], and indomethacin, one of the NSAIDs drug, has been found to inhibit PGE2 production with IC_50_ = 0.45 μM [32]. Although the IC_50_ value was not measured in this study, the anti-inflammatory activity of **1**–**3** was moderate at a concentration of 10 μM or higher compared with other anti-inflammatory agents in previous studies.

## 3. Discussion

The anti-inflammatory effects of *P. frutescens* using an in vivo model have been considered in several studies. Yuan and coworkers studied the effects of the *P. frutescens* extract against chronic obstructive pulmonary disease (COPD) airway inflammation in cigarette smoke/LPS-induced COPD mice [33]. They observed significant decreases in inflammatory cell infiltration in lung tissue and the production of inflammatory cytokines in the bronchoalveolar lavage fluid. Oh et al. also showed the protective effects of the *P. frutescens* var. *acuta* extract (EPPF) and **3** (rosmarinic acid, RA) against allergic inflammations in an ovalbumin (OVA)-sensitized mouse model [34]. In the OVA-sensitized mice, the number of nasal rubbings and the concentrations of IgE and histamine were decreased by EPPF or RA administration. In addition, the mRNA and protein levels of IL-1β, IL-6, and TNF-α were decreased after administering EPPF or RA in the OVA-sensitized mice. Along with **3**, various compounds have also been tested using in vivo anti-inflammatory models. For example, tormentic acid has shown comparable activity with hydrocortisone (ID_50_ = 0.03 mg/ear) in reducing inflammatory responses in a mouse model experiment [35].

Although clinical studies of *P. frutescens* extract and its constituents are scarce, a few noteworthy clinical trials have been conducted. For example, Kim et al. showed that eight weeks of *P. frutescens* extract intake can relieve pain and improve knee joint function in patients with knee joint pain [36]. In addition, RA (**3**, 200 mg or 50 mg per day for 21 days) was orally administered to patients with seasonal allergic rhinoconjunctivitis (SAR) and the study reported a reduction in the SAR symptoms, the concentrations of cytokine release, and the quantity of neutrophils/eosinophils in the nasal lavage fluid [37]. RA has been topically applied as a cream (0.3%) to atopic dermatitis (AD) patients, and the symptoms were shown to be mitigated [38]. Even though no clinical trial has been conducted with **1** and **2** to date, several scientific reports have demonstrated the beneficial effects of the compounds for eye fatigue [39,40].

As far as we know, this study reports the in silico simulation of a PPAR-α/δ/γ agonist, in vitro PPAR-α/δ/γ, and the NF-κB transcription activities of **1** and **2** for the first time. Although they did not increase PPAR-α/δ/γ promoter luciferase reporter activity, NF-κB transcriptional activity was inhibited by **1** and **2** dose-dependently, and **1** showed the most potent activity among the compounds. In addition, the mRNA expression levels of *Il6*, *Mcp1*, and *Tnfa* were suppressed by **1** and **2** in Raw 264.7 cells. The NF-κB pathway regulates the synthesis of pro-inflammatory cytokines, such as IL-6, MCP-1, and TNF-α. Degradation (phosphorylation) of IκBα by IκB kinase (IKK) disassembles the NF-κB p65 subunit from the complex and causes nuclear translocation of NF-κB to occur for an inflammatory response [41]. As compounds **1** and **2** were found to be effective NF-κB inhibitors in this study, further studies are required to elucidate the detailed mechanisms of action. For example, the phosphorylation/degradation of IκBα and the nuclear translocation of p65 by **1** and **2** will be conducted shortly [42].

Rosmarinic acid (**3**) inhibited NF-κB activation as a PPAR-α agonist, which was shown for the first time in this study. Although Rajagopal and coworkers have reported that **3** acts as a PPAR-γ agonist in a docking simulation study [43], the potential of **3** as a PPAR-α/δ agonist has first been shown in this study. It has been reported that **3** alleviated inflammation by suppressing the TGF-β/IL-17A pathway in human adipocytes [44], and also attenuated the inflammation of cardiomyocytes by initiating the PPAR-γ/NF-κB signaling pathway [30]. As demonstrated in this study, the activation of PPARs inhibits NF-κB-dependent inflammation; and the AMP kinase (AMPK)-SIRT1/p300 pathway was involved in the process [45,46,47]. In particular, PPAR activators promote AMPK, which increases SIRT1 expression and p300 phosphorylation. The activated AMPK-SIRT1/p300 signal leads to the decreased acetylation of the p65 subunit in the NF-κB complex and translocation into the nucleus, resulting in the reduced expression of NF-kB p65 target genes, such as *Il6, Il1b*, and *Tnfa*. In addition, PPAR-α/γ agonists potentiate IκBα expression, which is an inhibitory protein against NF-κB, and, thus, induce anti-inflammatory activity [48,49]. Activation of PPAR-δ also inhibits the assembly of TAK1, TAB1, and HSP27, consequently interfering with the function of p65 NF-κB [50].

A limitation of this study is that the in silico simulations of **1**–**3** as PPAR-α/δ/γ agonists showed inconsistencies with the results from the in vitro PPAR transcriptional assay. Indeed, major limitations may have been caused by the limited conformations of the ligand–receptor in pose prediction, the effects of the solvents, or the approximated scoring system [51]. Nevertheless, docking simulation helps to investigate the potential of compounds for therapeutic activities and to predict ligand/target relationships at a molecular level. A better description of the behavior of the ligand–receptor and refinement of the docking procedures will lead to a better correlation with the experimental data.

Although the protein levels of the pro-inflammatory cytokines could not be measured in this study, the genes measured in this study are not post-translationally regulated by phosphorylation or acetylation. Nicola et al. have reported that the mRNA expression of *Il6* and *Tnfa* is matched with the protein level in the serum [52], and the mRNA expression of *Mcp1* is also matched with the plasma concentration of MCP-1 [53].

## 4. Materials and Methods

### 4.1. Plant Material

The leaves of *P. frutescens* var. *acuta* were purchased from Megabiosoop in April 2019. A voucher specimen (No. EA387) has been deposited at the Natural Product Chemistry Laboratory, College of Pharmacy, Ewha Womans University. The perilla water extract was kept in a sterile bottle and refrigerated until further use for the isolation work and the in vitro assays.

### 4.2. General Experimental Procedures

Optical rotation was performed on a P-1010 polarimeter (Jasco, Tokyo, Japan), and the UV spectrum was recorded on a U-3000 spectrophotometer (Hitachi, Tokyo, Japan). The NMR spectrum was determined on a Varian Unity Inova 400 MHz FT-NMR instrument (Agilent Technologies, Santa Clara, CA, USA) with TMS as an internal standard, and the data were processed in MestReNova 9.0 (Mestrelab Research SL, Santiago de Compostela, Spain). HRESIMS was performed on an Agilent 6230 Accurate-Mass TOF LC/MS system (Agilent, Santa Clara, CA, USA). For column chromatography, Diaion HP-20 and Kieselgel 60 F254 (silica gel, 0.25 mm layer thickness) were purchased from Mitsubishi Chemical Co. (Tokyo, Japan) and Merck & Co. (Rahway, NJ, USA), respectively. MPLC was performed using CombiFlash (Teledyne Isco Inc., Lincoln, NE, USA), equipped with a RediSep Rf C18 column (130 g, Teledyne Isco Inc., Lincoln, NE, USA) and a RediSep Rf normal phase silica column (40 g and 220 g). Preparative HPLC purification was conducted using an Acme 9000 system (Young Lin, Seoul, Korea) equipped with a YMC-Pack Pro C18 column (5 μm, 250 mm × 20 mm i.d., YMC Co., Kyoto, Japan).

### 4.3. Extraction and Isolation

The dried leaves of *P. frutescens* var. *acuta* (2 kg) were extracted with water (20 L) for 15 h at room temperature, and then the extract was evaporated in vacuo at 40 °C to achieve a concentrated water extract (352.8 g). The water extract was chromatographed over Diaion HP-20 using a gradient mixture (MeOH-H_2_O, 0:100 to 100:0) to produce the pooled fractions (Fr.1-Fr.7). Fr.4 (37.7 g) was subjected to RP-MPLC (flow rate: 10 mL/min) with a mixture of MeOH-H_2_O (1:19 to 100:0) to give seven subfractions (Fr.4.1-Fr.4.7). Compound **1** (2.1 g, yield: 0.1050%*w*/*w*) was precipitated from Fr.4.2. A part (2.0 g) of Fr.6 (7.5 g) was subjected to RP-MPLC (flow rate: 10 mL/min) with a gradient mixture (MeOH-H_2_O, 1:19 to 100:0) and the subfraction Fr. 6.3 (125.9 mg) was purified using a preparative HPLC instrument with an isocratic solvent system (30% MeOH, 8 mL/min) to produce **2** (*t*_R_ 85.3 min, 49.4 mg, yield: 0.0025%*w*/*w*). Fr. 7 (7.9 g) was subjected to MPLC (flow rate: 5 mL/min) with a solvent mixture (CH_2_Cl_2_-MeOH, 100:0 to 0:100) to acquire 9 subfractions (Fr.7.1-Fr.7.9). Subfraction Fr.7.4.4.5.5 (252.6 mg) was purified on a preparative HPLC instrument using an isocratic solvent system (50% MeOH, 5 mL/min) to yield **3** (*t*_R_ 13.3 min, 27.6 mg, yield: 0.0014%*w*/*w*).

Luteolin 7-*O*-diglucuronide (**1**): yellow amorphous solid; [α]_D_^20^ −34.6 (c 0.1, MeOH); UV (MeOH) *λ*_max_ (log ε) 254 (4.68), 347 (4.67); HRESIMS m/z 639.1194 [M + H]^+^ (calcd for C_27_H_27_O_18_); ^1^H NMR (pyridine-*d*_5_, Figure S1) *δ*_H_ 7.86 (d, H-2′, *J* = 2.3 Hz), 7.46 (dd, H-6′, *J* = 2.3, 8.2 Hz), 7.23 (d, H-5′, *J* = 8.2 Hz), 7.18 (d, H-8, *J* = 2.0 Hz), 7.15 (d, H-6, *J* = 2.0 Hz), 6.83 (s, H-3), 6.04 (d, H-1″, *J* = 6. 8 Hz), 5.57 (d, H-1′″, *J* = 8.2 Hz), 4.92 (d, H-5″, *J* = 9.6 Hz), 4.75 (m, H-4″, H-5′″), 4.61 (m, H-2″, H-3″, H-4′″), 4.40 (t, H-3′″, *J* = 9.0 Hz), 4.27 (t, H-2′″, *J* = 8.2 Hz); ^13^C NMR (pyridine-*d*_5_, Figure S2) *δ*_C_ 182.8 (C-4), 172.6 (C-6′″), 172.0 (C-6″), 165.3 (C-2), 163.7 (C-7), 162.7 (C-5), 157.8 (C-9), 151.8 (C-4′), 147.7 (C-3′), 122.7 (C-1′), 119.7 (C-6′), 116.8 (C-5′), 114.7 (C-2′), 107.0 (C-1′″), 106.8 (C-10), 104.0 (C-3), 100.9 (C-6), 100.3 (C-1″), 95.9 (C-8), 84.2 (C-2″), 78.2 (C-5′″), 77.9 (C-3′″), 77.6 (C-5″), 77.0 (C-3″), 76.2 (C-2′″), 73.8 (C-4′″), 72.7 (C-4″) [39].

Apigenin 7-*O*-diglucuronide (**2**): white amorphous solid; [α]_D_^20^ −62.7 (c 0.1, MeOH); UV (MeOH) *λ*_max_ (log ε) 268 (4.60), 334 (4.66); HRESIMS m/z 623.1243 [M + H]^+^ (calcd for C_27_H_27_O_17_); ^1^H NMR (pyridine-*d*_5_, Figure S3) *δ*_H_ 7.83 (d, H-2′, H-6′, *J* = 7.4 Hz), 7.29 (d, H-8, *J* = 2 Hz), 7.20 (d, H-3′, H-5′, *J* = 7.4 Hz), 7.16 (d, H-6, *J* = 2 Hz), 6.81 (s, H-3), 6.09 (d, H-1″, *J* = 7.6 Hz), 5.57 (d, H-1′″, *J* = 8.4 Hz), 4.94 (d, H-5″, *J* = 9.5 Hz), 4.74 (t, H-4″,H-5′″, *J* = 9.5 Hz), 4.60 (m, H-2″, H-3″, H-4′″, *J* = 7.6 Hz), 4.40 (t, H-3′″, *J* = 9.1 Hz), 4.26 (m, H-2′″, *J* = 9.1,8.4 Hz); ^13^C NMR (pyridine-*d*_5_, Figure S4) *δ*_C_ 182.9 (C-4), 172.6 (C-6′″), 172.1 (C-6″), 164.9 (C-2), 163.8 (C-7), 162.8 (C-4′), 162.7 (C-5), 157.8 (C-9), 129.0 (C-2′ and C-6′), 116.8 (C-3′ and C-5′), 107.0 (C-1′″), 106.8 (C-10), 103.9 (C-3), 101.0 (C-6), 100.3 (C-1″), 95.9 (C-8), 84.2 (C-2″), 78.2 (C-5′″), 77.8 (C-3′″), 77.6 (C-5″), 77.1 (C-3″), 76.2 (C-2′″), 73.4 (C-4′″), 72.7 (C-4″) [39].

Rosmarinic acid (**3**): yellow amorphous solid; [α]_D_^20^ 101.3 (c 0.07, MeOH); UV (MeOH) *λ*_max_ (log ε) 328 (4.40); HRESIMS m/z 359.0767 [M-H]^-^ (calcd for C_18_H_17_O_8_, 360.0764); ^1^H NMR (methanol-*d*_4_, Figure S5) *δ*_H_ 7.54 (d, H-7, *J* = 15.8 Hz), 7.04 (d, H-2, *J* = 2.7 Hz), 6.94 (dd, H-6, *J* = 2.7,8.4 Hz), 6.77 (d, H-5, *J* = 8.4 Hz), 6.75 (d, H-2′, *J* = 1.8 Hz), 6.69 (d, H-5′, *J* = 8.2 Hz), 6.62 (d, H-6′, J = 1.8,8.2 Hz), 6.26 (d, H-8, *J* = 15.8 Hz), 5.17 (q, H-8′, *J* = 4.1, 8.8 Hz), 3.10 (dd, H-7′, *J* = 4.1, 14.3 Hz), 2.99 (dd, H-7′, *J* = 8.8, 14.3 Hz); ^13^C NMR (methanol-*d*_4_, Figure S6) *δ*_C_ 168.6 (C-9), 149.7 (C-4), 147.5 (C-7), 146.9 (C-3), 146.2 (C-3′), 145.2 (C-4′), 129.7 (C-1′), 127.8 (C-1), 123.1 (C-6), 121.8 (C-6′), 117.6 (C-2′), 116.5 (C-5), 116.3 (C-5′), 115.2 (C-2), 114.8 (C-8), 75.3 (C-8′), 38.2(C-7′) [54].

### 4.4. Molecular Docking

The crystal structures of PPAR-α/δ/γ were obtained from the RCSB PDB website [PDB ID: 1K71 (PPAR-α); 1GWX (PPAR-δ); and 3DZY (PPAR-γ)] (https://www.rcsb.org/, accessed on 16 March 2023). The 3D structures of **1**–**3** and EPA (a positive control) were acquired from the PubChem website (https://pubchem.ncbi.nlm.nih.gov/, accessed on 16 March 2023). Three programs were used for docking simulation: Autodock Vina 1.1.2 (Scripps Research, San Diego, CA, USA), Autodock 4.2.6 (Scripps Research, San Diego, CA, USA), and Dock6.10 (UCSF, San Francisco, CA, USA). Docking preparation was conducted to add hydrogens and assign charges of the compounds in UCSF Chimera (UCSF, San Francisco, CA, USA). Pharmacophore analysis was conducted using LigandScout 4.0 (Inte:Ligand, Vienna, Austria) to explore possible interactions of the receptors and ligands. ADMETlab 2.0 was used to perform ADMET prediction analysis for the compounds (**1**–**3**) [23].

### 4.5. Cell Viability

The cell viability of the Raw 264.7 macrophages was determined by 3-(4,5-dimethylthiazolyl-2)-2,5-diphenyl tetrazolium bromide (MTT) assay. At 70% confluence, Raw 264.7 cells were treated with PWE (0, 10, 50, 100 µg/mL) or **1**–**3** (0, 5, 10, 50 µM) for 24 h. After aspirating the cell culture medium, cells were incubated in DMEM with 10% FBS and 5 mg/mL MTT solution. After 1 h of incubation, the concentration of formazan, a purple product converted from a tetrazolium salt by the viable cells, was measured using a spectrophotometer at 595 nm.

### 4.6. PPAR and NF-κB Transcriptional Activity

Luciferase assays were performed to determine the transcriptional activity of the PPAR transcription factors in the Ac2F cell. Briefly, Ac2F cells were transfected with the PPRE-X3-TK-LUC plasmid (0.2 µg) with PPAR-α, PPAR-δ, or PPAR-γ expression vectors (0.1 µg) using Lipofectamine 3000 reagent (Invitrogen, Carlsbad, CA, USA). The cells were further treated with **1**–**3** or WY14643 (a known PPAR-α agonist), GW501516 (a known PPAR-δ agonist), and rosiglitazone (a known PPAR-γ agonist), respectively. The luciferase activity was measured using the One-Glo Luciferase Assay System (Promega, Madison, WI, USA). After adding the luciferase substrate, the luminescence was measured using a luminescence plate reader (Berthold Technologies GmbH & Co., Bad Wildbad, Germany).

Luciferase assays were also performed to determine the transcriptional activity of NF-κB in the HEK293T cells. The cells were transfected with the NF-κB promoter-Luc plasmid (0.1 µg) for 24 h, co-treated with test compounds **1**–**3** and LPS (1 µg/mL) for 6 h, and lysed using a One-Glo Luciferase Assay System and a luminescence plate reader. The results are presented as mean ± S.E. (*n* = 5), and each measurement was performed in triplicates. Statistical significance was tested using a one-way ANOVA/post hoc test.

### 4.7. NF-κB Target Gene Expression

Raw 264.7 macrophages (Korea Cell Line Bank, Seoul, Korea) were grown in DMEM containing 4500 mg/L glucose and L-glutamine (Sigma-Aldrich, St. Louis, MO, USA) supplemented with 10% FBS (Sigma-Aldrich) and 1% antibiotic antimycotic solution at 37 °C and 5% CO_2_. Two days after confluence, PWE (0, 10, 50, 100 µg/mL) or **1**–**3** (0, 5, 10, 50 µM) dissolved in DMSO (Sigma-Aldrich) were treated for 24 h, and with 100 ng/mL of LPS (Sigma-Aldrich) for 6 h. Then, total RNA was extracted from Raw 264.7 cells using Trizol reagent (Invitrogen, Carlsbad, CA, USA), and cDNA was synthesized from 1 μg of total RNA using the PrimeScript II 1st strand cDNA synthesis kit (Takara, Japan). The mRNA levels of *Il6*, *Mcp1*, and *Tnfa* were quantified using a StepOnePlus Real-time PCR System (Applied Biosystems, Waltham, MA, USA) and SYBR Green PCR Master Mix (Applied Biosystems, Waltham, MA, USA), and then normalized relative to 18S rRNA. The fold changes of gene expression were calculated by the ΔΔCt method. The specific primer sequences used are shown in Table S1.

## 5. Conclusions

In this study, two flavonoid diglucuronides, luteolin 7-*O*-diglucuronide (**1**) and apigenin 7-*O*-diglucuronide (**2**), and rosmarinic acid (**3**) were isolated from the leaves of *P. frutescens* var. *acuta*. In silico PPAR docking simulation unlocked the potential of **1**–**3** as PPAR agonists, and these results were evaluated using in vitro PPAR luciferase assay. In addition, PWE and **1**–**3** suppressed the LPS-induced upregulation of *Il6*, *Mcp1*, and *Tnfa* in Raw 264.7 cells, which may be mediated through the PPAR/NF-κB signaling pathway. This study provided evidence that the *P. frutescens* var. *acuta* water extract and the isolated compounds have anti-inflammatory activity. Further investigation will be directed to elucidate the detailed mechanisms of action, such as the phosphorylation/degradation of IκBα and the nuclear translocation of p65 by the compounds. Moreover, if possible, the in vivo anti-inflammatory activity and the pharmacokinetics of **1**–**3** will be investigated to evaluate the bioavailability and toxicity of the compounds as drug candidates.

## Figures and Tables

**Table 1:** Docking energy (Kcal/mol) of **1**–**3** and EPA with PPAR-α/δ/γ.

	Compound	Autodock Vina	Autodock 4	Dock6
PPAR-α	EPA *	−6.7	−7.8	−35.5
	**1**	−7.2	−13.2	−41.5
	**2**	−6.5	−9.1	−41.7
	**3**	−8.6	−9.7	−43.0
PPAR-δ	EPA	−7.8	−7.4	−41.0
	**1**	−9.7	−14.7	−58.1
	**2**	−9.4	−13.2	−43.3
	**3**	−8.3	−9.3	−41.5
PPAR-γ	EPA	−6.8	−8.1	−37.2
	**1**	−5.8	−9.7	−57.1
	**2**	−5.6	−13.4	−51.6
	**3**	−7.6	−8.9	−40.1

* A positive control.

**Table 2:** Results of ADMET prediction for **1**–**3**.

Category	Feature	EPA	1	2	3
Physicochemical properties	Molecular weight	302.22	638.11	622.12	360.08
	Van der Waals (Volume)	356.24	564.63	555.84	346.37
	Density	0.85	1.13	1.12	1.03
	No. ^a^ of H-bond ^b^ acceptors	2	18	17	8
	No. of H-bond donors	1	10	9	5
	No. of rotatable bonds	13	7	7	7
	No. of rings	0	5	5	2
	No. of atoms in the biggest ring	0	10	10	6
	Pure LogS (log mol/L)	−4.42	−4.37	−2.94	−2.95
	LogP	5.18	−0.31	0.88	1.51
Medicinal chemistry	SA score ^c^	3.04	4.88	4.74	2.90
	MCE-18	0	127.46	123.92	30
	Lipinski rule	Accepted	Rejected	Rejected	Accepted
	Pfizer rule	Rejected	Accepted	Accepted	Accepted
	GSK rule	Rejected	Rejected	Rejected	Accepted
Absorption	Caco-2 cell permeability (log unit)	−5.08	−6.90	−6.94	−5.80
	MDCK cell permeability (cm/s)	1.7 × 10^−5^	5.96 × 10^−5^	3.30 × 10^−5^	5.00 × 10^−6^
	F_20%_	0.94	0.01	0.84	0.98
Distribution	Plasma protein binding (%)	100.70	83.34	81.14	92.41
	Volume distribution (L/kg)	0.26	0.61	0.50	0.36
	BBB penetration probability	0.001	0.054	0.045	0.021
	Fu (The fraction unbound in plasma %)	1.09	14.64	11.64	3.31
Metabolism	CYP1A2-inhibition probability	0.072	0.116	0.041	0.251
	CYP1A2-substrate probability	0.117	0.015	0.006	0.022
	CYP2C19-inhibition probability	0.028	0.048	0.054	0.064
	CYP2C19-substrate probability	0.050	0.034	0.030	0.034
	CYP2C9-inhibition probability	0.116	0.004	0.004	0.481
	CYP2C9-substrate probability	1.00	0.09	0.15	0.94
Excretion	Clearance (mL/min/kg)	1.77	1.10	1.04	9.52
Toxicity	Human hepatotoxicity probability	0.92	0.14	0.25	0.59
	Ames toxicity probability	0.003	0.305	0.042	0.235
	Rat oral acute toxicity probability	0	0.008	0.027	0.272
	Carcinogenicity probability	0.105	0.037	0.109	0.536
	Respiratory toxicity probability	0.535	0.014	0.012	0.034
Toxicophore rules	Acute toxicity rule (alerts)	0	0	0	0
	Genotoxic carcinogenicity rule (alerts)	0	0	0	1
	Non-genotoxic carcinogenicity rule (alerts)	0	0	0	1
	SureChEMBL rule (alerts)	0	0	0	0

^a^ Number; ^b^ Hydrogen bond; ^c^ Synthetic accessibility score.

## Data Availability

Data is contained within the article and Supplementary Material.

## Supplementary Materials

The following supporting information can be downloaded at: https://www.mdpi.com/article/10.3390/ph16121655/s1, Figure S1. ^1^H NMR spectrum of compound **1**; Figure S2. ^13^C NMR spectrum of compound **1**; Figure S3. ^1^H NMR spectrum of compound **2**; Figure S4. ^13^C NMR spectrum of compound **2**; Figure S5. ^1^H NMR spectrum of compound **3**; Figure S6. ^13^C NMR spectrum of compound **3**; Table S1. Primer sequences used for quantitative real-time PCR; Table S2. The cell viability (%) of Raw 264.7 cells using an MTT assay; Table S3. PPAR-α agonistic potency of **1**–**3** compared to that of the control; Table S4. PPAR-δ agonistic potency of **1**–**3** compared to that of the control; Table S5. PPAR-γ agonistic potency of **1**–**3** compared to that of the control; Table S6. Inhibition of NF-κB transcriptional activity compared to that of the control; Table S7. Inhibition of NF-κB transcriptional activity by the *Perilla* extract and **1**–**3**.
