# Supplementary material for: Anti-Inflammatory Activity of the Constituents from the Leaves of Perilla frutescens var. acuta
Source: Pharmaceuticals (Basel). 2023 Nov 28;16(12):1655. doi: 10.3390/ph16121655 (PMC10747482; doi:10.3390/ph16121655)
Supplement: Supplementary file 1 [file pharmaceuticals-16-01655-s001.zip › pharmaceuticals-2717022-supplementary.pdf]

Anti-inflammatory activity of the constituents from the leaves of *Perilla frutense* var. *acuta*

Isoo Youn [1,†], Sujin Han [1,†], Hee Jin Jung [2], Sang Gyun Noh [2], Hae Young Chung [2], Yeon Kyoung Koo [3], Sunhye Shin [4,*], and Eun Kyoung Seo [4,*]

[1] Graduate School of Pharmaceutical Sciences, College of Pharmacy, Ewha Womans University, Seoul 03760, Korea; isooyoun87@gmail.com (I.Y.); sujinh94@gmail.com (S.H.); yuny@ewha.ac.kr (E.K.S.)
[2] Department of Pharmacy, College of Pharmacy, Pusan National University, Busan 46241, Korea; hjjung2046@pusan.ac.kr (H.J.J); rskrsk92@pusan.ac.kr (S.G.N.); hyjung@pusan.ac.kr (H.Y.C.)
[3] Department of R&I Center, COSMAXBIO, Seongnam 13487, Korea; ygkoo@cosmax.com (Y.K.K.)
[4] Major of Food and Nutrition, Division of Applied Food System, Seoul Women's University, Seoul 01797, Korea; ygkoo@cosmax.com (Y.K.K.)
[*] Correspondence: yuny@ewha.ac.kr (E.K.S.); sshin@swu.ac.kr (S.S.)
[†] These authors contributed equally to this work.

TABLE OF CONTENTS

Figure S1. ^{1}H NMR spectrum of compound **1**……………………………………………………………3

Figure S2. ^{13}C NMR spectrum of compound **1**……………………………………………………...………3

Figure S3. ^{1}H NMR spectrum of compound **2**……………………………………………………...……….4

Figure S4. ^{13}C NMR spectrum of compound **2**………………………………...…………………………4

Figure S5. ^{1}H NMR spectrum of compound **3**……………………………………………………...……….5

Figure S6. ^{13}C NMR spectrum of compound **3**…………………………………...……………………5

Table S1. Primer sequences used for quantitative real-time PCR……………………………...…………6

Table S2. The cell viability (%) of Raw 264.7 cells using an MTT assay…………………..……………….6

Table S3. PPAR-α agonistic potency of **1-3** compared to that of the control…………………..……………6

Table S4. PPAR-δ agonistic potency of **1-3** compared to that of the control…………………..……………6

Table S5. PPAR-γ agonistic potency of **1-3** compared to that of the control……………………..…………6

Table S6. Inhibition of NF-κB transcriptional activity compared to that of the control……………….………7

Table S7. Inhibition of NF-κB transcriptional activity by the Perilla extract and **1-3**…………………….……7

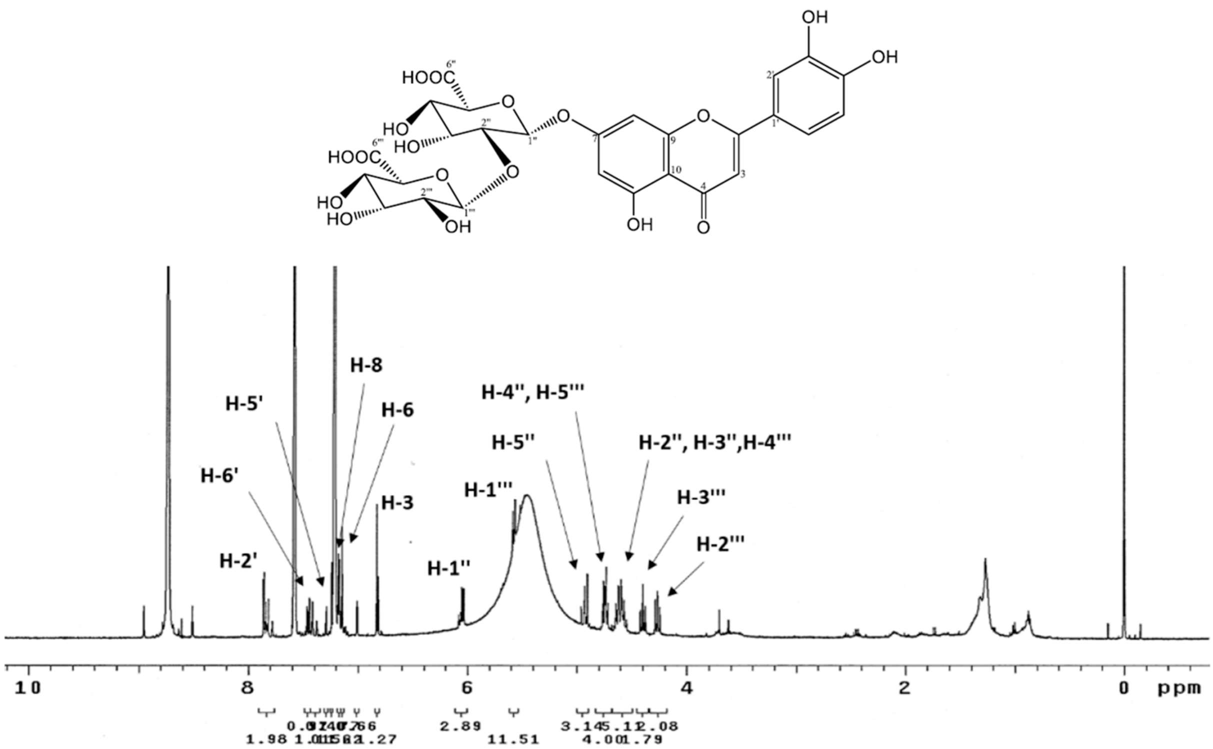

Figure S1. ^{1}H NMR spectrum of compound **1** (pyridine-d_5 as solvent)

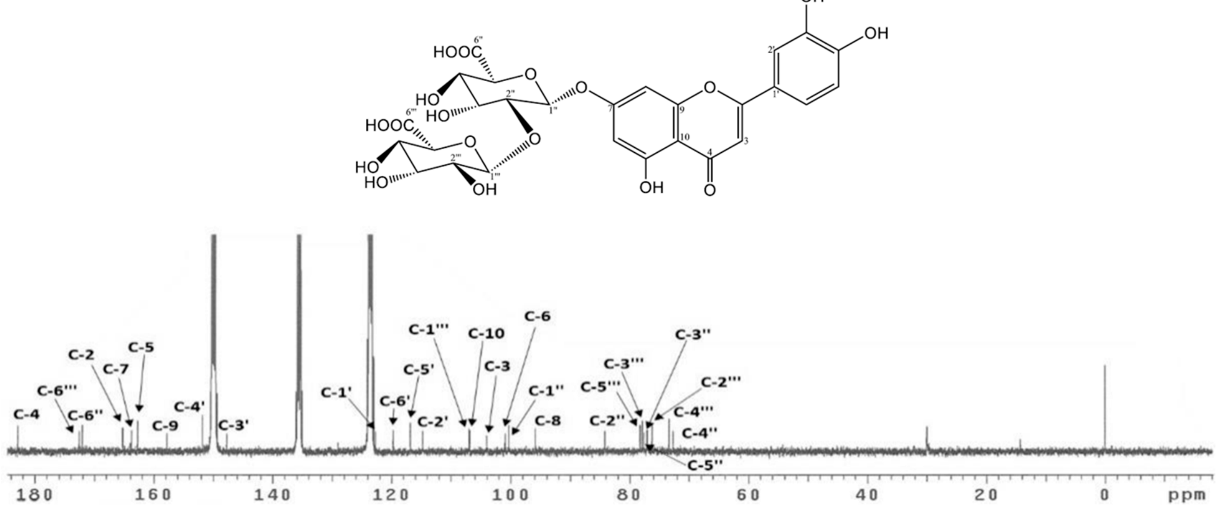

Figure S2. ^{13}C NMR spectrum of compound **1** (pyridine-d_5 as solvent)

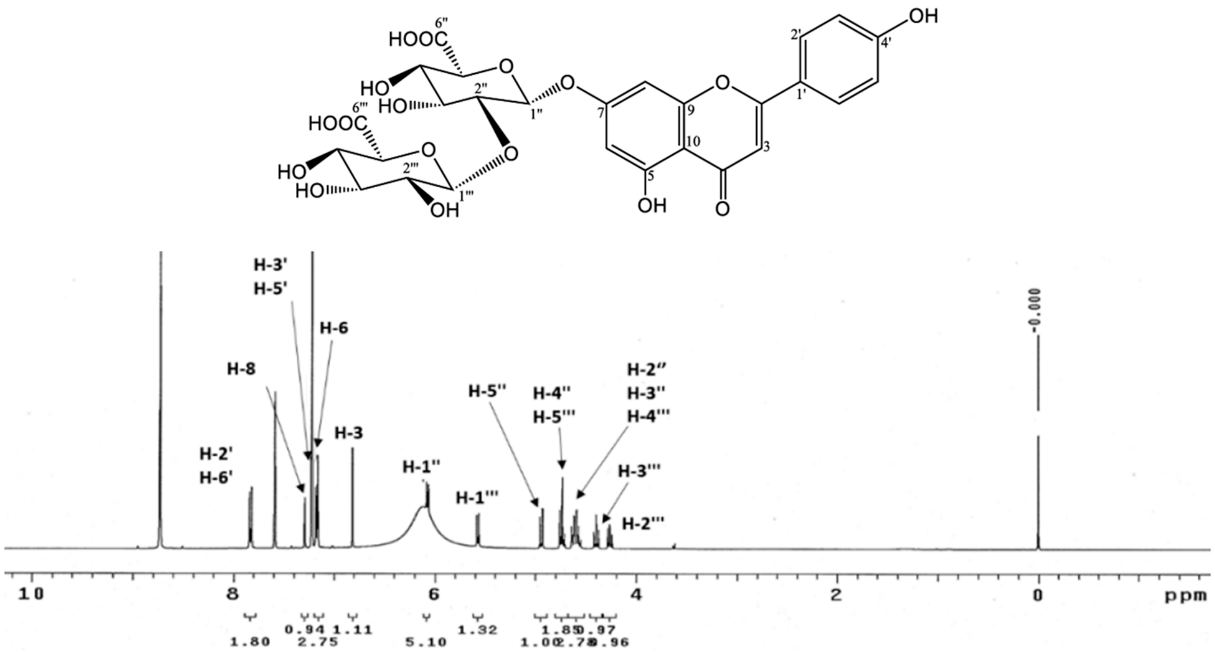

Figure S3. ^{1}H NMR spectrum of compound **2** (pyridine-d_5 as solvent)

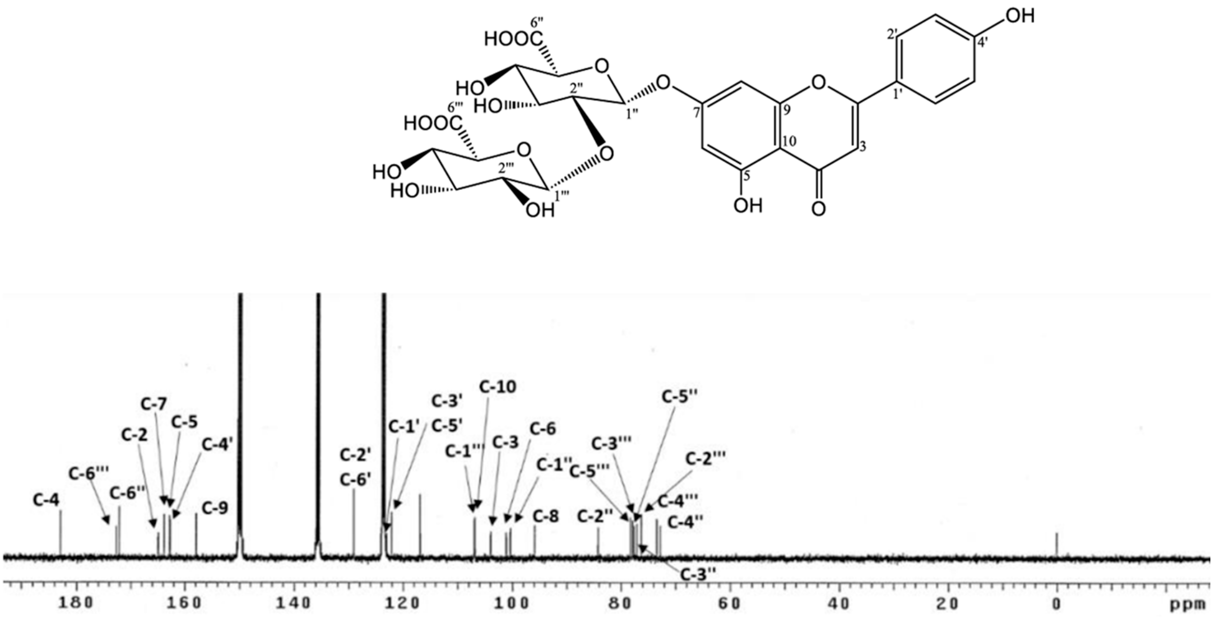

Figure S4. ^{13}C NMR spectrum of compound **2** (pyridine-d_5 as solvent)

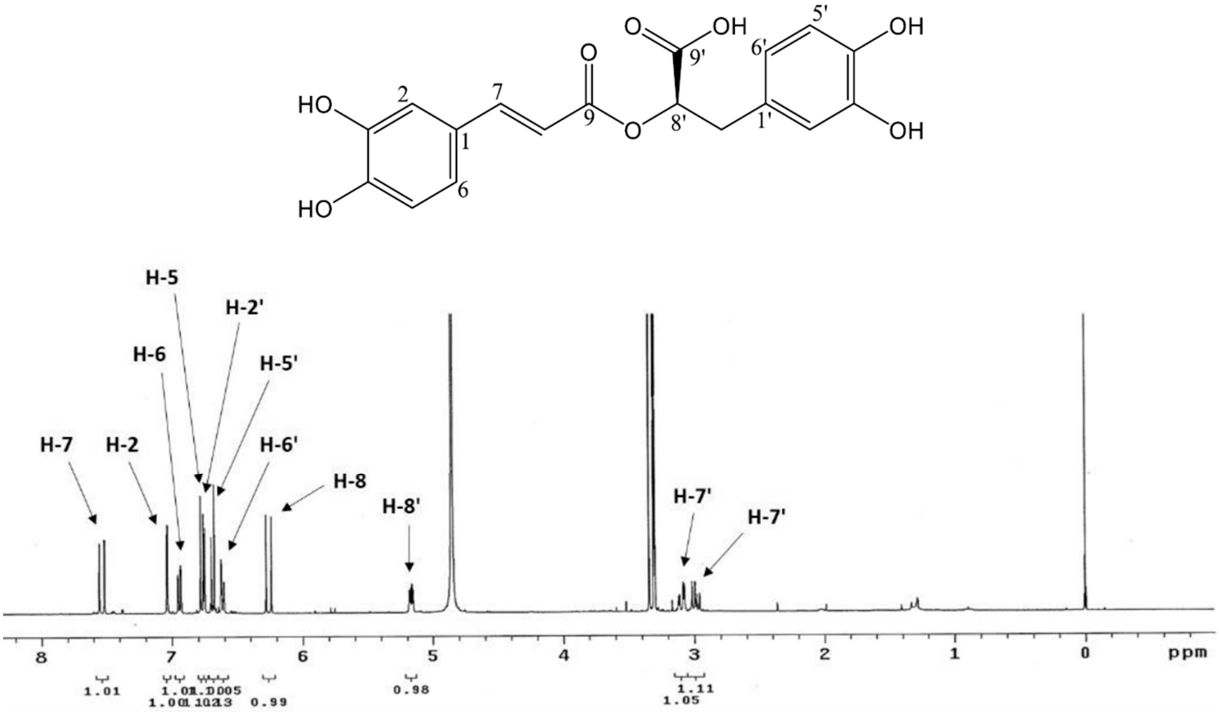

Figure S5. ^{1}H NMR spectrum of compound **3** (methanol-d_4 as solvent)

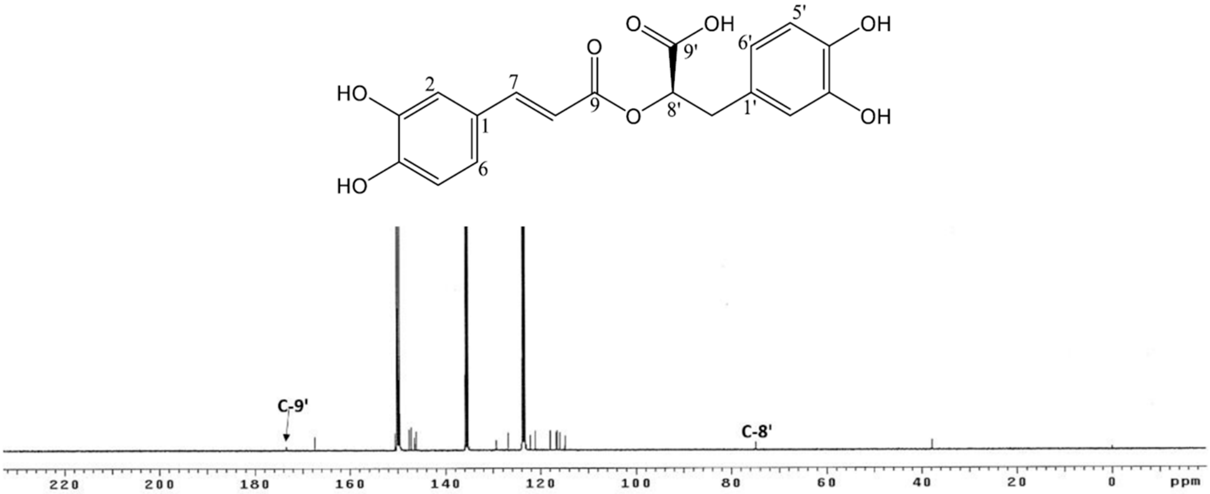

Figure S6. ^{13}C NMR spectrum of compound **3** (methanol-d_4 as solvent)

Table S1. Primer sequences used for quantitative real-time PCR.

Gene	Forward Primer	Reverse Primer
18S	ATC CCT GAG AAG TTC CAG CA	CCT CTT GGT GAG GTC GAT GT
Il6	TTC CTC TCT GCA AGA GAC TTC C	TGA AGT CTC CTC TCC GGA CTT
Mcp1	CAC TCA CCT GCT GCT ACT CA	GCT TGG TGA CAA AAA CTA CAG C
Tnfa	AGC CCC CAG TCT GTA TCC TT	CTC CCT TTG CAG AAC TCA GG

Table S2. The cell viability (%) of Raw 264.7 cells using an MTT assay.

**Extract**				**1**		**2**		**3**	
Conc. (μg/mL)	CV[a] (%)	Stdv[b]	Conc. (μM)	CV[a] (%)	Stdv[b]	CV[a] (%)	Stdv[b]	CV[a] (%)	Stdv[b]
0	100.0	4.0	0	100.0	0.5	100.0	4.2	100.0	8.5
10	97.5	2.0	5	111.4	2.9	94.7	2.2	105.7	1.4
50	80.3	2.7	10	114.6	3.3	98.7	1.1	100.8	2.5
100	81.8	0.8	50	111.0	12.8	94.3	1.9	100.6	3.0

[a]Cell viability. [b]Standard deviation.

Table S3. PPAR-α agonistic potency of **1-3** compared to that of the control.

**Blank**	PPAR-α + PPRE[a]	WY14643	Extract[b]	**1**[c]	**2**[c]	**3**[c]
Percentage (%)	100.00	158.36	170.93	133.30	121.61	160.58
ratio	1.00	1.58	1.71	1.33	1.22	1.61

[a]A Control. [b]A concentration of the extract: 20 μg/mL. [b]A concentration of **1-3**: 10μM.

Table S4. PPAR-δ agonistic potency of **1-3** compared to that of the control.

**Blank**	PPAR-δ + PPRE[a]	GW501516	Extract[b]	**1**[c]	**2**[c]	**3**[c]
Percentage (%)	100.00	143.36	118.84	91.23	80.34	101.92
Ratio	1.00	1.43	1.19	0.91	0.80	1.02

[a]A control. [b]A concentration of the extract: 20 μg/mL. [b]A concentration of **1-3**: 10μM.

Table S5. PPAR-γ agonistic potency of **1-3** compared to that of the control.

**Blank**	PPAR-γ + PPRE[a]	Rosiglitazone	Extract[b]	**1**[c]	**2**[c]	**3**[c]
Percentage (%)	100.00	153.79	106.44	101.85	117.22	88.58
Ratio	1.00	1.54	1.06	1.02	1.17	0.89

[a]A control. [b]A concentration of the extract: 20 μg/mL. [b]A concentration of **1-3**: 10μM.

Table S6. Inhibition of NF-κB transcriptional activity compared to that of the control.

		1[b]			2[b]			3[b]		
	LPS[a]	5 μM	10 μM	50 μM	5 μM	10 μM	50 μM	5 μM	10 μM	50 μM
Percentage (%)	100.00	43.17	25.04	20.14	56.66	36.42	28.86	44.14	38.79	24.72
Inhibition (%)	0.00	56.83	74.96	79.86	43.34	63.58	71.14	55.86	61.21	75.28

[a]A control (1 μg/mL). [b]Three concentrations of **1-3** were 5, 10, and 50 μM.

Table S7. Inhibition of NF-κB transcriptional activity by the Perilla extract and **1-3**.

	**Extract**				**1**		
Conc.	*Il6*/*18s*	*Mcp1*/*18s*	*Tnfa*/*18s*	Conc.	*Il6*/*18s*	*Mcp1*/*18s*	*Tnfa*/*18s*
**0 μg/mL**	0.00	0.00	0.00	0 μM	0.00	0.00	0.00
10 μg/mL	-17.74	45.96	37.57	5 μM	-38.76	-39.62	3.74
50 μg/mL	0.69	56.95	61.44	10 μM	-28.72	-44.71	4.32
100 μg/mL	24.14	63.27	77.24	50 μM	79.00	67.94	49.09
	**2**				**3**		
Conc.	*Il6*/*18s*	*Mcp1*/*18s*	*Tnfa*/*18s*	Conc.	*Il6*/*18s*	*Mcp1*/*18s*	*Tnfa*/*18s*
0 μM	0.00	0.00	0.00	0 μM	0.00	0.00	0.00
5 μM	-36.21	-69.95	6.09	5 μM	7.20	30.69	27.74
10 μM	-30.89	-44.39	19.34	10 μM	28.56	53.88	39.56
50 μM	80.97	44.65	21.98	50 μM	31.50	35.74	31.81
